# Does volume change of the spleen correlate with the therapy response in uveal melanoma patients with liver metastases undergoing hepatic artery infusion chemotherapy?

**DOI:** 10.2478/raon-2025-0047

**Published:** 2025-09-05

**Authors:** Hannah Luisa Steinberg-Vorhoff, Marcel Drews, Marcel Opitz, Natalie van Landeghem, Luca Salhöfer, Mathias Holtkamp, Yan Li, Johannes Haubold, Jens Siveke, Heike Richly, Michael Forsting, Benedikt Michael Schaarschmidt, Sebastian Zensen

**Affiliations:** Institute of Diagnostic and Interventional Radiology and Neuroradiology, University Hospital Essen, Essen, Germany; Institute for Developmental Cancer Therapeutics, West German Cancer Center, University Hospital Essen, Essen, Germany; Division of Solid Tumor Translational Oncology, German Cancer Consortium (DKTK, partner site Essen) and German Cancer Research Center, DKFZ, Heidelberg, Germany; Department of Medical Oncology, Sarcoma Center, West German Cancer Center, University of Duisburg-Essen, Essen, Germany

**Keywords:** hepatic artery infusion chemotherapy, uveal melanoma, spleen size, survival, liver metastases

## Abstract

**Background:**

Uveal melanoma (UM) patients with liver metastases often undergo hepatic artery infusion therapy (HAIC). Due to diffuse metastatic spread in the liver, patients often develop hepatomegaly and secondary, portal hypertension which may lead to splenomegaly. This study aimed to compare spleen volumetry and the change of spleen volume (SV) for the evaluation of HAIC treatment response.

**Patients and methods:**

In this study, 179 UM patients (mean age 64.8 ± 11.0y, 53% female) with liver metastases undergoing HAIC were included. Treatment response was analyzed by RECIST 1.1 and SV on CT imaging before and after first HAIC. The correlation of change in spleen and liver volume was analyzed with Spearman test. Overall survival (OS) was calculated as the time from the first HAIC to patient death using Kaplan-Meier test and multivariate analysis was performed for RECIST 1.1 and SV.

**Results:**

In the study population, OS was 13.8 months (95% CI 10.6-14.7 months). Change in SV before and after first HAIC was +4% (interquartile range [IQR] -4.0%–12.0%, p = 0.49) and showed a weak correlation with OS (r = -0.11, p = 0.18). UM patients with progressive disease (PD) according to RECIST 1.1 showed an increase in SV compared to patients with stable disease (SD) (p = 0.04). Compared to RECIST 1.1, SV was not significant prognostic factor that can identify a change in OS.

**Conclusions:**

In uveal melanoma patients with liver metastases undergoing HAIC, neither the change of SV nor splenomegaly could be identified as prognostic factors for OS.

## Introduction

Uveal melanoma (UM) is the most common primary malignant disease of the eye and accounts for approximately 5% of all melanomas.^1,2^ The local tumor can be treated aggressively. However, around 50% of all UM patients develop metastases, in approximately 60% of patients liver metastases.^3,4^ There is no consensus regarding the treatment of liver metastases in UM patients.^5^ The only systemic immune therapy with a prolonged OS for UM patients is tebentafusp. A phase 3 study by Hassel *et al*. showed that OS in UM patients with liver metastases treated with tebentafusp was prolonged to 21.6 months compared to the control group with an OS of 16.9 months.^6^ However, tebentafusp is only eligible for HLA-A*02:01-positive patients. For all other UM patients, there is no systemic therapy superior to another.^7^ In this palliative setting, liver-directed therapies such as transarterial chemoembolization (TACE), radioembolization (RE), or hepatic artery infusion chemotherapy (HAIC) have proven to be well-tolerated treatment options.^8–10^ Therefore, minimizing side effects is crucial and HAIC is particularly noteworthy. Particularly, repeated HAIC is a well-tolerated therapy option that showed prolonged progression-free survival and fewer hematological severe adverse events compared to intravenous chemotherapy.^11^ Monitoring the response of liver metastases to therapy is crucial for optimizing patient care and outcome. Metastases of various tumor histologies can induce hepatomegaly due to their progressive growth.^12^ Hepatomegaly is defined with a volume larger than 1890 ml.^13^ Hepatomegaly secondary to diffuse metastatic spread to the liver can, in turn, lead to portal hypertension and splenomegaly. The growth of the spleen can be explained not only by the increased portal pressure but also by the spleen’s role in the immune system. The spleen, as part of the immune system and the largest lymphoid organ, plays a significant role in tumor progression.^14^ For hepatocellular carcinoma (HCC), it has been demonstrated that post-hepatectomy splenomegaly may indicate liver decompensation and the presence of splenomegaly with portal vein thrombosis is associated with a shorter overall survival (OS).^15,16^ Splenomegaly can be easily detected in computed tomography (CT) and magnetic resonance imaging (MRI) and is defined as a spleen volume greater than 315 ml.^17^ To date, no studies have investigated the correlation between splenomegaly, the change in volume of the spleen, and therapy response in UM patients with liver metastases.

Therefore, the aim of this study was to analyze the correlation between changes in spleen and liver volume, the relevance of splenomegaly and therapy response in UM patients with liver metastases undergoing HAIC.

## Patients and methods

### Patient cohort

**FIGURE 1.**
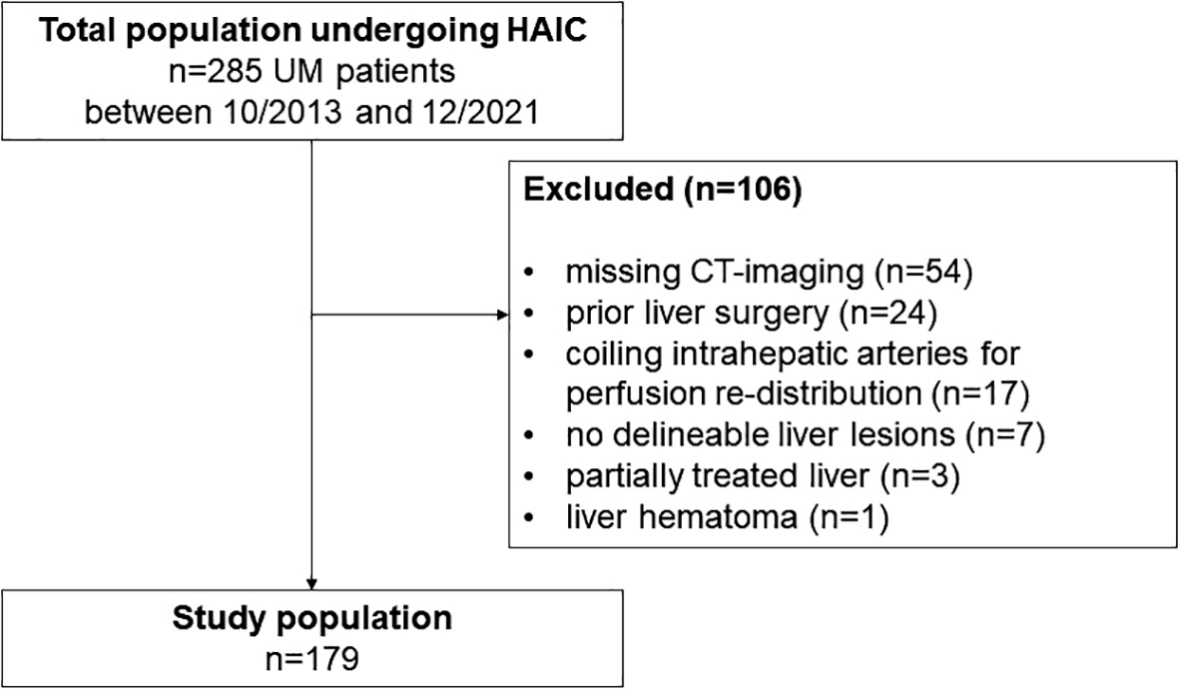
Flowchart of analyzed study population with exclusion criteria. HAIC = hepatic artery infusion therapy

In this single-center, retrospective study, UM patients who underwent their first HAIC for the treatment of liver metastases between January 2013 and December 2021 were included. Exclusion criteria were the lack of CT imaging before or after first HAIC, liver surgery before first HAIC, coiling of intrahepatic arteries for perfusion re-distribution, no delineable liver lesions, partially liver treated, or liver hematoma (Figure 1). Patient data were obtained from the medical record system. Survival data from all patients were obtained from the corresponding resident registration office. Ethical approval was granted by the local ethics committee, and the requirement to obtain informed consent was waived (19-8703-BO, approval date 26.07.2019). The study was conducted in compliance with the guidelines of the Institutional Review Board of the University Hospital Essen.

### Hepatic artery infusion chemotherapy (HAIC) procedure

HAIC was performed as described by Heusner *et al*.^9^ In the angiography suite, the femoral artery was punctured, and a 5F sheath was inserted using Seldinger technique. Then, the upper abdominal arteries were catheterized using a 5F Sidewinder catheter, and a hepaticography was performed. A microcatheter was coaxially advanced and placed first in the common hepatic artery, then in the right and left hepatic artery, and selective angiographies were performed. When necessary, extrahepatic vessels originating from the hepatic arteries or accessory hepatic vessels were catheterized by a microcatheter and occluded with coils. Depending on the flow dynamics and tumor distribution, the drug was infused either into both lobes simultaneously via the proper hepatic artery or both lobes were treated separately via the right and left hepatic artery. Patients started with a dose of 40 mg of Melphalan, and in case of progression, the dose was escalated up to 50 mg, or the chemotherapeutic agent was switched to Fotemustin.

### Volumetry and therapy response assessment

**FIGURE 2.**
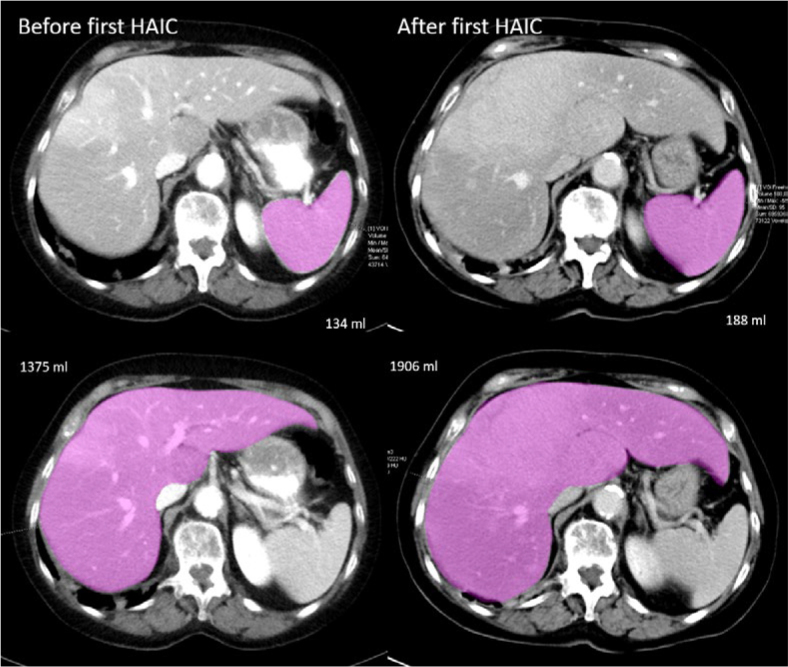
71-year old women with uveal melanoma and liver metastases. Axial CT image examples of volumetry of liver and spleen four days before and 44 days after first hepatic artery infusion therapy (HAIC). Liver and spleen volume increased by 39% after first HAIC.

Volumetry of the liver and spleen was performed in consensus by two radiologists with several years of experience in oncological CT imaging using syngo. via (Siemens Healthineers, Germany, Figure 2). CT examinations of the liver were performed in arterial and portalvenous contrast phases. CT examinations were performed a maximum of two weeks before and 6–8 weeks after first HAIC. The liver index lesions were measured in the same contrast phase before and after HAIC in which they could be most sharply delineated. At least one index lesion was selected within the liver and the longitudinal diameter was measured, or if at least one index lesion per lobe was delineable, the diameters were summed up. In accordance with RECIST 1.1, therapy response was categorized into stable disease (SD), progressive disease (PD), and partial response (PR).^18^ OS was defined as the time from first HAIC to death. In order to investigate the influence of a change in spleen volume on OS, we chose a threshold value of +5% after first HAIC to differentiate stable (decrease or increase ≤ +5%) from increase (> +5%) of spleen volume. A threshold of 5% was chosen because it exceeds the typical range of inter-observer and technical variability in imaging-based spleen measurements and represents a meaningful change of size.

### Statistics and data analysis

D’Agostino-Pearson-Test was applied to determine normal distribution. Normally distributed data are reported as mean ± standard deviation, non-normally distributed data as median and interquartile range (IQR). The Kaplan-Meier curves and the log-rank test were applied to estimate OS with 95% confidence intervals (95% CI). Mann-Whitney-U test was used to compare differences in non-normally distributed groups. Spearman test was applied for correlation in non-normally distributed data. For survival analysis, cox regression analysis was used. A p-value ≤ 0.05 was considered statistically significant. Statistical analysis was performed using GraphPad Prism 10.0.2 (GraphPad Software, San Diego, USA).

## Results

### Patient and treatment characteristics

A total of 285 UM patients with liver metastases underwent first HAIC in the study period. Of these, 179 patients (62.8%) with a mean age of 64.8 years (SD 11.0, range 27–84 years) could be included in this study (Table 1). Female were 53.1% (95/179) of patients. The median number of HAICs per patient was 5 (IQR 3–10). The median time between the baseline and follow-up CT after first HAIC was 6.9 weeks (IQR 6.3–7.6 weeks). Median time between baseline CT and first HAIC was 1 day (IQR 1–3 days, range 0–13 days). 91.6% (164/179) of patients were treated with 40 mg Melphalan at first HAIC, while 3.4% (6/179) received 45 mg, 1.7% (3/179) received 50 mg and 1.1% (2/179) received 35 mg, 2.2% (4/179) of patients were treated with 40 mg Melphalan and 450 mg degradable starch microspheres (DSM) (EmboCept S, PharmaCept, Berlin, Germany) for salvage therapy due to extensive liver metastases. According to RECIST 1.1 criteria, 82.7% (148/179) of patients were evaluated SD after first HAIC, while 16.8% (30/179) were evaluated PD and 0.6% (1/179) PR.

**TABLE 1.** Patient characteristics

	All patients (n = 179)	Splenomegaly (n = 25)	No Splenomegaly (n = 154)	p-value
Age (mean/SD)	64.8 years, 11.0 years	61.9 years, 12.5 years	65.0 years, 10.7 years	0.2
Gender (female/male)	95 (53 %) / 84 (47 %)	4 (16 %) / 21 (84 %)	91 (59 %) / 63 (41 %)	
Number of HAIC per patient	5 (3–10)	5 (3–11)	5 (3–9)	0.8
Spleen				
Volume before first HAIC	205 ml (150–263 ml)	362 ml (347–407 ml)	186 ml (142–237 ml)	< 0.0001
Volume after first HAIC	213 ml (155–279 ml)	385 ml (345–430 ml)	200 ml (151–241 ml)	< 0.0001
Volume change	+4.0 % (-4.0 – +12.0%)	+ 6.5 % (-5.8 – +11.5%)	+3.6 % (-3.4 – +12.2%)	0.8
Liver				
Volume before first HAIC	1731 ml (1426–2228 ml)	2358 ml (1936–2787 ml)	1637 ml (1405–2050 ml)	< 0.0001
Volume after first HAIC	1780 ml (1447–2406 ml)	2354 (1983–2894 ml)	1741 ml (1431–2240 ml)	0.0009
Volume change	+4.0 % (-2.0 – +13.0%)	-0.5 % (-9.4 – +6.9%)	+5.0 % (-1.7 – +13.4%)	0.04
RECIST 1.1 liver index size change	+2.8 % (-6.5 – +14.3%)	+2.5 % (-3.0 – +10.2%)	+3.3 % (-7.2 – +16.1%)	0.7
Overall survival	13.7 months (7.3–21.2 months)	10.7 months (5.2–19.2 months)	13.8 months (7.3–21.8 months)	0.6

1 Values are shown as median (IQR), if not otherwise specified.

1 HAIC = hepatic artery infusion therapy; SD = standard deviation

Median spleen volume before first HAIC was 205 ml (IQR 150–263 ml, range 51–605 ml), which slightly increased after first HAIC to 213 ml (IQR 155–279 ml, range 65–662 ml), resulting in a median change of +4.0% (IQR -4.0% – +12.0%, p = 0.49). Accordingly, to a threshold of 315 ml, 14.0% (25/179) of patients presented with splenomegaly, and 86.0% (154/179) without splenomegaly before first HAIC. Patients with splenomegaly more often presented with hepatomegaly and, consequently, with significant higher liver volume before first HAIC (median 2358 ml, IQR 1936–2787 ml) compared to those without splenomegaly (median 1637 ml, IQR 1405–2050, p < 0.0001). In the entire study population, the median liver volume before first HAIC was 1731 ml (IQR 1426–2228 ml, range 889–7116 ml), which increased to 1780 ml (IQR 1447–2406 ml, range 1447–7078 ml) after first HAIC, resulting in a median change of +4.0% (IQR -2.0% – +13.0%, p = 0.26). The change spleen volume following first HAIC did not significantly differ regardless of the presence of splenomegaly (with splenomegaly: 6.5%, IQR -5.8 – +11.5%, without splenomegaly: 3.6%, IQR -3.4 – +12.2%; p = 0.8). Conversely, the liver volume in patients without splenomegaly increased significantly more after first HAIC compared to patients with splenomegaly (with splenomegaly: -0.5%, IQR -9.4 – +6.9%; without splenomegaly: 5.0%, IQR 1.7–13.4%; p = 0.04). The median change in RECIST 1.1 liver index lesion size was +2.8% (IQR -6.6% – +14.3%, range -86.2% – +134.2%). There is a moderate correlation between change in size of the spleen and the liver (r = 0.34 p < 0.0001), as well as between change in size of the liver and liver index lesion (r = 0.32, p < 0.0001). Change in size of the spleen and liver index lesion showed a weak correlation (r = 0.2, p = 0.009).

**FIGURE 3.**
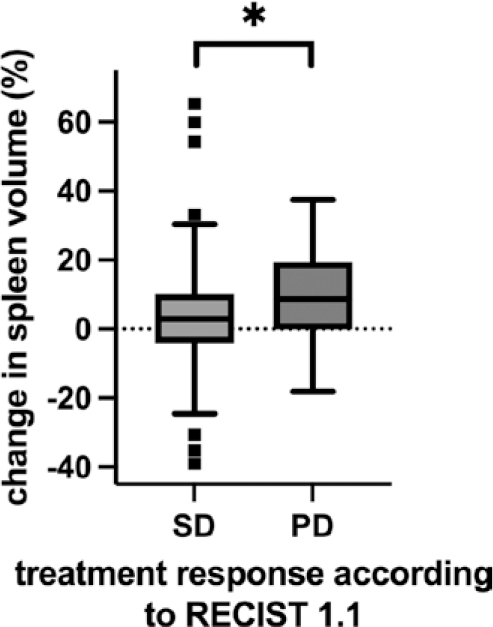
Change in spleen volume dif ferentiated by treatment response according to RECIST 1.1. Points show outliers outside the Tukey whiskers. Asterisks indicate significant differences (p ≤ 0.05).

Patients with SD had prior to first HAIC a median spleen volume of 204 ml (IQR 151–264 ml) and increased after first HAIC to 213 ml (IQR 157–277 ml). Patients with PD had prior first HAIC a median spleen volume of 212 ml (IQR 134–247 ml) and after first HAIC a median spleen volume of 213 ml (IQR 130–281 ml). There is no significant difference in spleen volume between patients with SD compared to patients with PD prior first HAIC (p = 0.34) and after first HAIC (p = 0.60). While patients with SD had a median change in spleen volume of 2.9% (IQR -4.1% – +10.2%), patients with PD had a higher median change in spleen volume of 8.6% (IQR -0.16% – +19.4%, p = 0.04, Figure 3).

### Survival analysis

**FIGURE 4.**
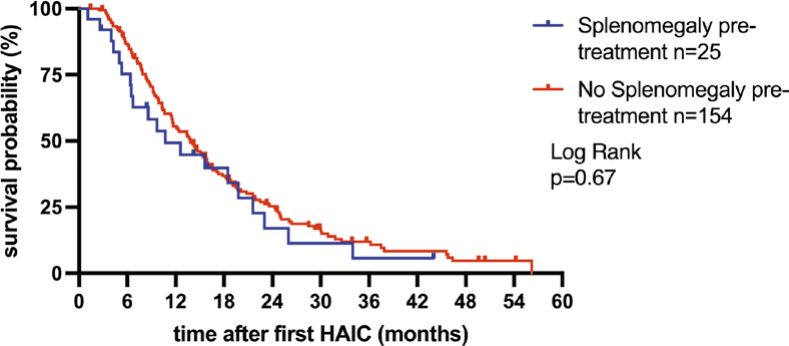
Kaplan-Meier curve shows overall survival (OS) in uveal melanoma (UM) patients with and without splenomegaly before first hepatic artery infusion therapy (HAIC).

**FIGURE 5.**
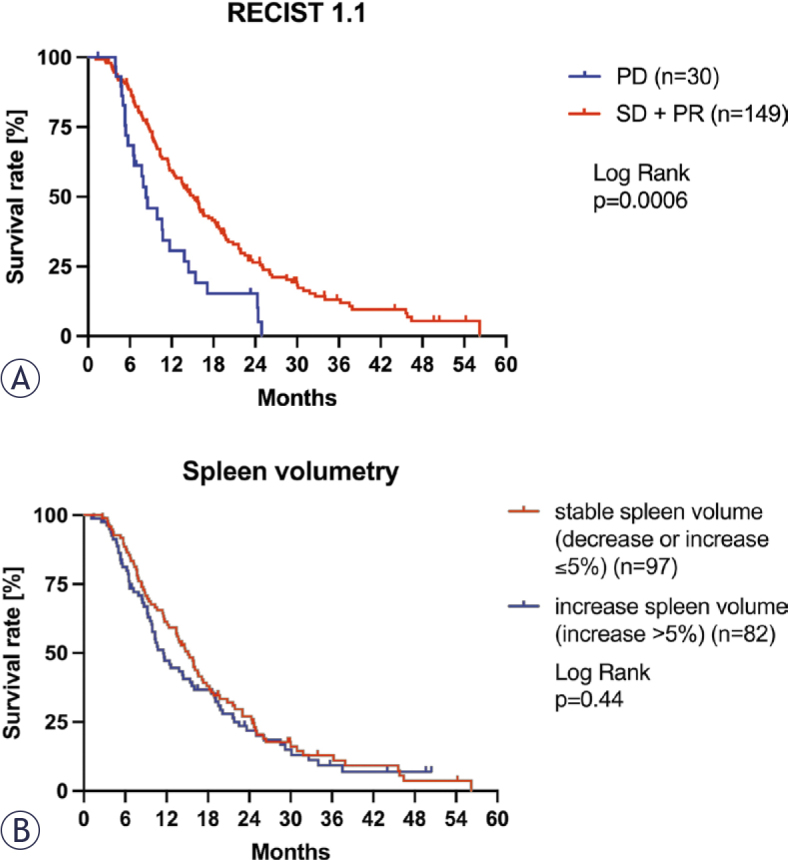
Overall survival of evaluation of treatment response by RECIST 1.1 **(A)** and spleen volumetry with a threshold of 5% **(B)** of spleen volume increase of uveal melanoma patients with liver metastases treated by hepatic artery infusion therapy (HAIC). Kaplan-Meier curves show overall survival (OS) separately for patients evaluated as stable disease (SD) + partial response (PR) and (progressive disease) PD. In spleen volumetry, patients with an increase in spleen volume more than 5% were classified as increased spleen volume and with decrease or increase up to 5% as stable spleen volume.

Before the date of analysis 83.8% (150/179) of patients died, while 16.2% (29/179) were alive or lost to follow-up. Median OS of the study population was 13.8 months (95% CI 10.6–14.7 months). There is no significant difference in the median OS between patients with and without splenomegaly before first HAIC (with splenomegaly: 10.7 months, 95% CI 6.4–15.9 months; without splenomegaly: 13.8 months, 95% CI 11.4–15.4 months, p = 0.41, Figure 4). Change in spleen volume and OS showed a very weak negative correlation in the entire study population (r = -0.11, p = 0.18), as well as in the subgroups with splenomegaly (r = 0.15, p = 0.48) and without splenomegaly (r = -0.13, p = 0.15). OS was significantly shorter in patients with PD according to RECIST 1.1 (8.3 months, 95% CI 5–14 months, n = 30) compared to patients with SD or PR (15.1 months, 95% CI 8–22 months, n = 149; Chi square 11.8, p = 0.0006, Figure 5A). In spleen volumetry, there is no significant difference in OS in the patient group with increasing spleen volume (15.1 months, 95% CI 8.1–21.9 months; n = 97) and stable spleen volume (11.5 months, 95% CI 6.4–19.9 months; n = 82, Chi square 0.61, p = 0.44, Figure 5B).

**FIGURE 6.**
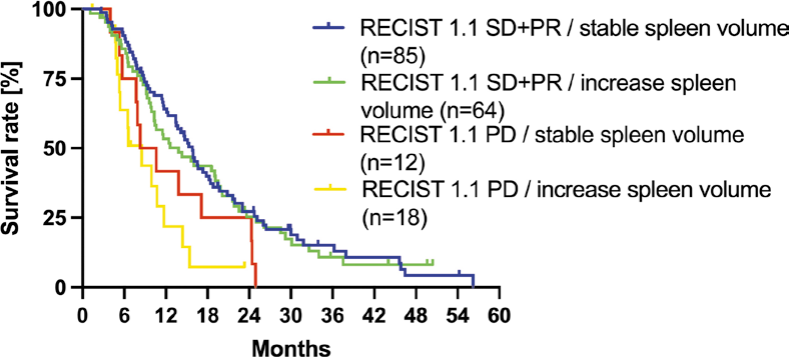
Kaplan-Meier curves for the combined RECIST 1.1 and spleen volumetry evaluation of treatment response with a threshold of 5% increase in spleen volume in uveal melanoma patients with liver metastases treated by HAIC. PD = progressive disease; PR = partial response; SD = stable disease

There was a slight agreement of spleen volume with RECIST 1.1 according to the inter-rater reliability analysis (κ = 0.005, 95% CI: -0.046 – 0.0056; Table 2). We further analyzed RECIST 1.1 and spleen volume in combination with no significant result (Figure 6). There is no significant difference in OS in patients with SD+PR according to RECIST 1.1 and either increase of or stable spleen volume (RECIST 1.1 SD/increase spleen volume OS 13.8 months, 95% CI 7.4–22.3 months, RECIST 1.1 SD/stable spleen volume 15.8 months, 95% CI 8.5–21.9 months, Chi square 0.11, p = 0.74). Similarly, for patients with PD according to RECIST 1.1 and either increase of or stable spleen volume, the results are consistent (RECIST 1.1 PD/increase spleen volume OS 8.5 months, 95% CI 5.0–11.0 months, RECIST 1.1 PD/stable spleen volume 9.5 months, 95% CI 6.2–22.5 months, Chi square 1.46, p = 0.23).

**TABLE 2.** Median overall survival and accordance of treatment response evaluation by RECIST 1.1 and spleen volumetry with a threshold of 5% increase in spleen volume

Spleen volumetry			
RECIST 1.1 criteria	Stable (spleen volume decrease or increase up to max. 5%)	Increase (spleen volume increases more than 5%)	Total
SD + PR	15.8 months (n = 85)	13.8 months (n = 64)	15.1 months (n = 149)
PD	9.5 months (n = 12)	8.5 months (n = 18)	8.3 months (n = 30)
Total	15.1 months (n = 97)	11.5 months (n = 82)	13.8 months (n = 179)

1 Cohen ‘s κ = 0.005, 95% CI: -0.046 – 0.0056

1 PD = progressive disease; PR = partial response; SD = stable disease

## Discussion

In the present study, we analyzed the correlation between volume changes of the spleen and the relevance of splenomegaly for the therapy response in UM patients undergoing HAIC.

Our findings can be summarized into four main points. Firstly, changes in the volume of the spleen before and after first HAIC did not correlate with OS. Secondly, we found that splenomegaly before first HAIC was not predictive of shortened OS in UM patients undergoing HAIC. Thirdly, we observed a correlation between changes in spleen and liver volume before and after first HAIC. Lastly, patients with PD showed a significant increase in spleen size compared to patients with SD.

Since UM patients often develop liver metastases with a poor prognosis with a median OS of 4–6 months and a 1-year survival of 12–15% when left untreated, this approach is interesting to evaluate and could predict the treatment respons.^3,4,19^ There are several therapy options nowadays that prolong median overall survival after diagnosis of liver metastases to 13.4 months and a 2-year survival of 8%, which was similar in our study cohort.^4,20,21^ As there is no treatment superior to another, it is essential to understand the factors influencing treatment response and prognosis in this population for optimizing therapeutic strategies and improving patients’ outcom.^4,20–22^

To date, literature regarding the prognostic value of splenomegaly for patients undergoing HAIC is scarce. Former studies have shown that splenomegaly has a prognostic effect in patients with primary liver tumors treated with TACE, checkpoint inhibitors, and radiofrequency ablation.^15,23–25^ These results on primary liver tumors stand in contrast to our study, in which we could not identify pretreatment splenomegaly as a prognostic factor in UM patients undergoing HAIC. This could be due to various reasons. Our patient cohort, comprising only 25 patients with pre-treatment splenomegaly, was relatively small and may have been underpowered to detect subtle prognostic differences. In our department, the chemotherapeutic dosage was adapted dependent on the extent of liver metastases with patients with extensive liver metastases receiving a higher dosage, which could potentially confound the results. Furthermore, the discrepancy may be attributed to differences in tumor biology and treatment approaches between UM and primary liver tumors.

There are several studies that analyzed the change in volume of the spleen in correlation to the OS in patients receiving immunotherapy, and the results do not match. Galland *et al*. analyzed patients with non-small lung cancer (NSCLC) and came to the conclusion that the change in volume of the spleen correlates with the OS.^26^ In contrast to this result are the studies study by Castagnoli *et al*. that analyzed patients with NSCLC and the study by Müller *et al*. that analyzed hepatocellular carcinoma (HCC) patients and could not identify the change in spleen volume as a prognostic factor in overall survival.^27,28^ These results are similar to our study results. However, it has to be taken into consideration that the changes in volume of the spleen may be attributed not solely to treatment effects, but also to the immunotherapeutic interventions themselves since the spleen plays a major role in immune system.^14^ These examples show that further investigation is needed to understand the effect of change in spleen volume in tumor patients.

Unsurprisingly, our study shows that UM patients with PD have a shorter OS than patients with SD according to RECIST 1.1. The following analysis was conducted under the assumption that patients with progressive liver metastases develop portal hypertension, which can consequently lead to splenomegaly. We analyzed whether stable or increasing spleen volume within UM patients with liver metastases classified according to RECIST 1.1 could provide an additional estimation of survival probability. However, our findings did not reveal a significant correlation. It is important to note that the lack of significance may be attributed to the limited sample size of our cohort. A larger patient cohort, extension of the observation periods or different observation times of the spleen volume could potentially lead to different results.

Our study showed a correlation between changes in spleen and liver volume before and after first HAIC, as well as between the change in liver volume and change in liver index lesion. The liver may enlarge when there is diffuse and progressive metastatic spread leading to hepatomegaly and, consequently, to portal hypertension.^12^ As the liver undergoes changes in tumor burden and parenchymal alterations in response to TACE, it may exert indirect effects on spleen volume.

Lastly, our findings support the hypothesis that hepatomegaly, possibly caused by diffuse liver metastases, is associated with splenomegaly. This is demonstrated by the significant difference in liver volume observed in patients with splenomegaly compared to those without, prior to first HAIC. Furthermore, patients with PD, and consequently, a higher increase in liver volume showed a significant increase in spleen volume compared to patients with SD after first HAIC. This underlines the fact that growth in liver and spleen size are interconnected and diffuse metastatic spread of the liver may lead to portal hypertension.^29^

Our study has some limitations. Due to its retrospective and single-center study design, there may be selection bias in the patient cohort and the sample size did not allow a more detailed subgroup analysis. Furthermore, there are no portal hypertension direct measurements analysed in this study. Another limitation of this study is the heterogeneity of chemotherapy regimens (Melphalan 35–50 mg, DSM salvage), which may have influenced the change of spleen volume independently of tumor progression. Potential confounders of splenic size variability such as infections or concurrent medications were not accounted for. Nonetheless, the study cohort provides a unique insight into the importance of the size and change of size of the spleen in UM patients undergoing HAIC. Future research is necessary to evaluate the potential prognostic relevance of splenomegaly and the change of spleen volume in larger, longitudinal studies.

In UM patients undergoing HAIC, the change of spleen volume correlates with the change of liver volume. However, neither splenomegaly nor the change of spleen volume could be identified as prognostic factors for median OS.
